# Neurokinin-1 Receptor Antagonists as a Potential Novel Therapeutic Option for Osteosarcoma Patients

**DOI:** 10.3390/jcm12062135

**Published:** 2023-03-09

**Authors:** Prema Robinson, Marisa Rosso, Miguel Muñoz

**Affiliations:** 1 Department of Infectious Diseases, Infection Control and Employee Health, The University of Texas MD Anderson Cancer Center, Houston, TX 77030, USA; 2 Research Laboratory on Neuropeptides (IBIS), Neonatology Unit, Virgen del Rocío University Hospital, 41012 Seville, Spain; 3 Research Laboratory on Neuropeptides (IBIS), Pediatric Intensive Care Unit, Virgen del Rocío University Hospital, 41012 Seville, Spain

**Keywords:** osteosarcoma, apoptosis, antitumor, anti-metastasis, antiangiogenesis, NK-1 receptor, substance P

## Abstract

Osteosarcoma is a bone tumor predominantly affecting children and adolescents with high malignant potential. It is a cause of serious public health challenges due to its high morbidity rates and metastatic potential. Metastasis in osteosarcoma may manifest either during treatment of the primary tumor, shortly after treatment, or a long time after the end of the treatment. So far, there are no therapeutics that can prevent or treat osteosarcoma metastasis. The peptide substance P (SP) and its high-affinity receptor, Neurokinin-1 (NK-1R), are known to positively correlate with osteosarcoma progression. Osteosarcoma cells overexpress NK-1R. SP is known to elicit the proliferation of osteosarcoma cells and induce angiogenesis and migration, leading to the invasion and metastasis of tumor cells. In contrast, NK-1R antagonists, such as aprepitant, inhibit the proliferation and induce the apoptosis of osteosarcoma cells. Aprepitant is also known to inhibit the migration of osteosarcoma cells, as well as reduce the expression levels and activities of transcriptional regulators of metastasis-related genes such as matrix metalloproteinases (MMP-2 and MMP-9), vascular endothelial growth factor (VEGF), and nuclear factor kappa B (NF-κB). These preceding studies highlighted the antimetastatic role of aprepitant in osteosarcoma Moreover, combination therapy consisting of chemotherapy and NK-1R antagonist increases the chemosensitization of osteosarcoma cells. Interestingly, this combination therapy in vitro and in vivo decreases the severe side-effects of chemotherapy and produces neuroprotection, hepatoprotection, nephroprotection, and cardioprotection. In this review, we provide an update on existing data and suggest the need to repurpose aprepitant for use as an antitumor drug for treatment of osteosarcoma, and they suggest the need for phase I and II clinical trials for assessment of its safety/efficacy.

## 1. Introduction

Osteosarcoma is a highly malignant bone tumor and is the third most common cause of malignancy in children and adolescents. Although survival rates have increased up to 70% in patients with localized disease, the 5 year overall survival rate for patients with metastatic disease is below 30% [1,2]. So far, there are no therapeutics that can prevent or treat osteosarcoma metastasis. The current treatment of osteosarcoma includes surgery with pre- and postoperative chemotherapy. The histological response to preoperative chemotherapy is one of the most important criteria for prediction of clinical outcome of osteosarcoma. A cutoff of 90% histological necrosis has been shown to have the best prognostic value, while lower threshold values are not associated with significant survival benefits [3]. Chemotherapy displays two important limitations: chemoresistance and severe side-effects (cardiotoxicity, nephrotoxicity, hepatotoxicity, and neurotoxicity) [4]. Thus, it is imperative to discover novel agents that can effectively (a) prevent and treat metastasis, and (b) when combined with chemotherapy, be able to induce osteosarcoma chemosensitization and prevent severe side-effects of chemotherapy in osteosarcoma patients.

Substance P (SP) and the NK-1R system are widely known to positively correlate with cancer progression (e.g., osteosarcoma, melanoma, retinoblastoma, hepatoblastoma, glioma, rhabdoid tumors, acute myeloid leukemia, and lung carcinoma) [5,6,7,8,9,10,11,12]. Osteosarcoma cells express NK-1R to a significantly greater degree than nontumor cells [5] After binding to the NK-1R, SP regulates the activity of osteosarcoma cells (e.g., tumor cell proliferation/migration and antiapoptotic effects). In contrast, NK-1R antagonists counteract all these effects [5,13].

This review highlights NK-1R as being an important therapeutic target and outlines important studies that highlight the potentiality of NK-1R antagonists, such as aprepitant as a possible therapeutic agent in osteosarcoma patients.

## 2. SP/NK-1R System in Osteosarcoma 

SP, via interaction with NK-1R, promotes proliferation and prevents apoptosis of cancer cells, stimulates angiogenesis, and induces migration (leading to invasion/infiltration/metastasis of cancer cells) [14]. Osteosarcoma cells exhibit overexpression of both *TACR1* gene and NK-1R protein compared to nontumor cells [5]. The expression levels for the *TACR1* gene were compared between nontumor cells such as normal human fibroblasts and normal kidney epithelial cells, HEK293, and the osteosarcoma tumor cell line, MG-63. Relative gene expression levels for NK-1R in the HEK293 cells were only 50% of those observed in MG-63 cells, while human fibroblasts expressed only marginal amounts of NK-1R [5]. Similar results corelating the expression of mRNA with protein levels were demonstrated in these studies [5]. Important studies have shown that NK-1R is preferentially essential for the viability of cancer cells but not essential for the viability of normal nontumor cells [15]. Additionally, the role of the SP/NK-1R pathway in osteosarcoma is highlighted by studies showing SP to induce osteosarcoma cells to proliferate in a concentration-dependent manner [5,13] These preceding studies highlight NK-1R as being an important, potentially effective and selective therapeutic target for osteosarcoma. 

Neuropeptidases such as neutral endopeptidase (NEP) and dipeptidyl peptidase 4 (DPP-4) are enzymes that hydrolyze SP [16,17]. NEP (CD10) and DPP-4 (CD26) decrease the SP level. Moreover, reduced NEP may promote peptide-mediated proliferation by allowing the accumulation of higher peptide concentrations at the cell surface, thereby facilitating the development or progression of neoplasia [18]. CD10 is a favorable prognostic marker for childhood acute lymphoblastic leukemia. CD10-positive acute lymphoblastic leukemia cells have been identified as cycling cells with elevated c-myc levels and propensity to apoptosis, whereas CD10-negative acute lymphoblastic leukemia cells have lower cycling capacity and c-myc levels, with resistance to apoptosis in vitro [19]. Similarly, DPP-4 inhibitors have been shown to increase the risk of incidence of lung cancer as a result of their ability to increase the level of SP [20].

However, it has been reported that both NEP (CD10) and DPP-4 (CD26) are expressed at higher levels in human primary osteosarcoma bone tissues compared to adjacent noncancerous bone tissue, and that their combined expression correlates with poor prognosis [21]. Importantly, SP has been shown to play a very important role in osteosarcoma and other cancers. SP was shown to be a mitogen in osteosarcoma cells, while an NK-1R antagonist, aprepitant, counteracts the effects of SP, via the inhibition of proliferation and induction of apoptosis of osteosarcoma tumor cells. Furthermore, plasma levels of SP were shown to be higher in patients with cancer compared to healthy subjects [22]. SP is a mitogen in ALL and AML, while an NK-1R antagonist, aprepitant, was shown to inhibit ALL and AML cells and induce apoptosis in ALL and AML cells [11,12]. However, further studies in osteosarcoma should be carried out to clarify the contradictory results regarding the increase in SP-hydrolyzing enzymes NEP (CD10 and CD26) and their role in the pathogenesis of osteosarcoma.

Thus, the role of the SP/NK-1R pathway in osteosarcoma can be summarized as follows: (a) SP plasma levels are higher in cancer patients; (b) SP stimulates osteosarcoma cell proliferation in a concentration-dependent manner; (c) osteosarcoma cells overexpress NK-1R and NK-1R antagonists in a concentration-dependent manner, inhibiting proliferation and inducing apoptosis of osteosarcoma cells [13]. These above studies insinuate that one of the key mechanisms via which the SP/NK-1R system (that is overexpressed in cancer) may contribute to osteosarcoma pathogenesis is via activating NK-1R, thereby leading to SP-induced osteosarcoma cell proliferation and prevention of apoptosis. In contrast, specific NK-1R antagonists counteract osteosarcoma cell proliferation and induce osteosarcoma cell apoptosis. 

## 3. NK-1R Antagonists as Anticancer Drugs: A Therapeutic Approach against Osteosarcoma: Mechanistic Insights

### 3.1. Role of NK-1R Antagonists as Antiproliferative and Proapoptotic Agents in Osteosarcoma

**Figure 1:**
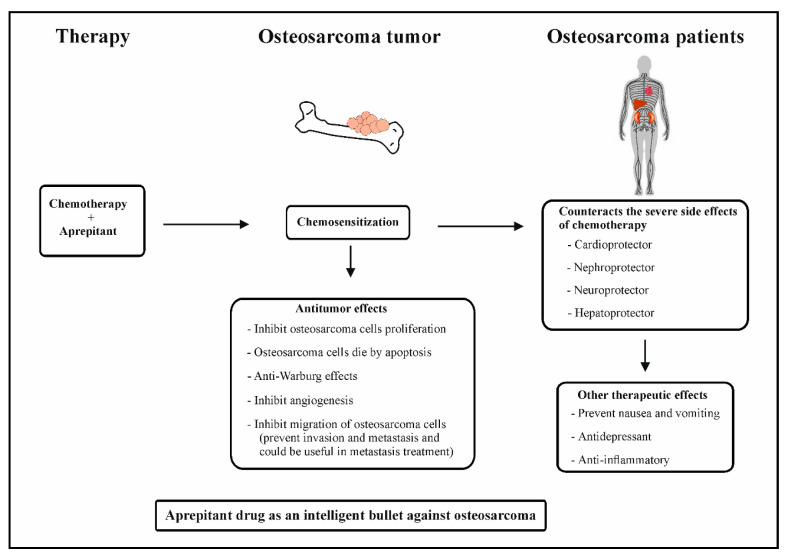
Combination of chemotherapy and aprepitant in osteosarcoma patients.

There are three NK-1R antagonists that have shown activity in the treatment of osteosarcoma in vitro and in vivo: L-733,060, L-732,138, and FDA-approved aprepitant for the treatment of chemotherapy-induced nausea and vomiting (CINV) [5]. The above NK-1R antagonists inhibit proliferation and induce apoptosis of osteosarcoma cells in a concentration-dependent manner [5,13] (Figure 1). Importantly L-733,060 was shown to decrease tumor volume in a murine preclinical model of osteosarcoma [5]. Positive Ki-67 expression was demonstrated to be associated with stage, distant metastasis, and overall survival of osteosarcoma, thus implicating its potential as a biomarker to predict prognosis and guide clinical therapy for osteosarcoma [23]. Similarly, in a hepatoblastoma xenograft mouse model, treatment with NK-1R antagonist aprepitant at 80 mg/kg/day for 24 days led to a significant reduction in tumor volume and weight, as well as in Ki-67-positive cells [8]. These studies highlight the potential of the NK-1R antagonist aprepitant to decrease tumor volume and the expression of Ki-67, as well as rationalize the potential need for future phase I and II clinical trials to be conducted to assess the potential of NK-1R antagonists, such as aprepitant, in osteosarcoma. 

### 3.2. Role of NK-1R Antagonists as Antiangiogenic Agents in Osteosarcoma

Neoangiogenesis, with resultant endothelial cell proliferation, new vessel formation, and increased blood flow, is a hallmark of tumor development. Angiogenesis has been shown to play a critical role in osteosarcoma progression and metastasis [5,13]. SP and NK-1R have been found in the intra- and peritumor blood vessels in a large majority of tumors [24]. It has also been reported that SP can directly stimulate the process of angiogenesis, through induction of endothelial cell proliferation. Two selective NK-1R antagonists have been shown to block SP-induced angiogenesis [8,25]. With specific relevance to osteosarcoma cells, aprepitant has been shown to decrease the expression of vascular endothelial growth factor (VEGF), a widely known mediator of angiogenesis [13] (Figure 1). Thus, the above findings suggest that aprepitant therapy can counteract tumor angiogenesis in osteosarcoma.

### 3.3. Role of NK-1R Antagonists as Agents That Counteract the Warburg Effect in Osteosarcoma

Glucose metabolism is strikingly different between normal cells and cancer cells. Production of ATP from glucose in normal cells leads to glucose oxidative phosphorylation via the TCA cycle. On the other hand, tumor cells convert glucose to lactate, which is known as the Warburg effect [26]. This landmark discovery has been the subject of great interest and has resulted in the study of various agents that target glucose metabolism. The metabolic shift of glucose to lactate in cancer cells results in the generation of less ATP per glucose molecule metabolized. To compensate for this reduced energy and the need for higher energy by rapidly multiplying cancer cells, there is a need for increased rates of glucose uptake via potential overexpression of glucose transporters (GLUTs and SLC2 gene family). The GLUT1 protein and gene are overexpressed in osteosarcoma cells. Most importantly, high levels of GLUT1 are significantly associated with lymph node metastasis, age, and low survival rate [27]. Furthermore, GLUT1 knockout studies have demonstrated significantly lower proliferation of OS cells in vitro and in vivo [28,29]. Moreover, in a nude mouse xenograft model of human osteosarcoma, the combination of adriamycin with a glycolytic inhibitor, 2-deoxy-D-glucose (2DG), resulted in significantly slower tumor growth (and, therefore, longer survival) compared to mice not treated with the glycolytic inhibitor [30]. 

Tumor cells predominantly produce energy by means of a high rate of glycolysis followed by lactic acid fermentation; this is known as the Warburg effect. SP, in a concentration-dependent manner, promotes the breakdown of glycogen into glucose in glioma cells; resulting in higher glucose levels compared to normal cells. NK-1R antagonists in glioma cells prevent high levels of glucose from being formed and the ensuing glycolysis, thereby preventing the Warburg effect [31,32]. These important preceding studies indicated that NK1-R antagonists may serve as potential drugs that can counteract the Warburg effect (Figure 1). Since osteosarcoma cells overexpress NK-1R, the use of an NK-1R antagonist, aprepitant, could counteract glucose production in osteosarcoma cells, thus preventing the Warburg effect in osteosarcoma cells. It is of tremendous importance that there are so far no drugs to counteract the survival effects of these tumor cells.

### 3.4. Role of NK-1R Antagonists as Antimetastatic Agents in Osteosarcoma

**Figure 2:**
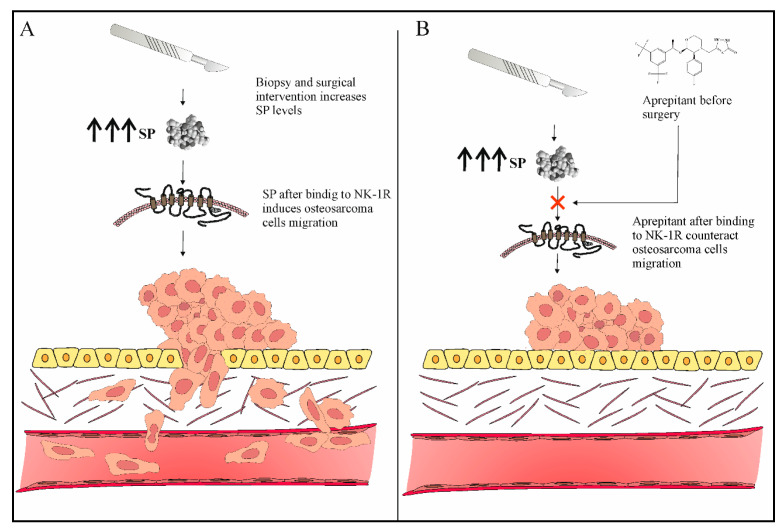
(**A**) Surgical procedures (biopsy or surgical intervention) can induce osteosarcoma invasion and metastasis. (**B**) Aprepitant therapy before surgery can prevent osteosarcoma invasion and metastasis.

Osteosarcoma very often manifests with the invasion and metastasis of osteosarcoma tumor cells, leading to poor prognosis [2]. Osteosarcoma metastasis may manifest during treatment of the primary tumor, shortly after treatment, or a long time after the end of the treatment [2]. Studies have determined that aprepitant has the ability to inhibit the migrative ability of osteosarcoma tumor cells and reduce the levels of matrix metalloproteinases (MMP-2 and MMP-9), as well as NF-κB, a known stimulator of metastasis-related genes [13]. Importantly, angiogenesis is one of the key factors leading to osteosarcoma progression and metastasis. Angiogenesis plays a critical role in osteosarcoma progression and metastasis. VEGF is the main angiogenic factor in tumor angiogenesis, supporting tumor growth and metastasis [33]. It has been shown that VEGF and MMP-9 expression in osteolytic lesions of bone is associated with a higher risk of local recurrence and bone destruction [34]. Aprepitant has the ability to reduce the levels of VEGF, thereby leading to reduced tumor metastasis [13]. The antimetastatic effect of aprepitant in osteosarcoma is probably attributed to its ability to modulate the transcriptional regulator nuclear factor kappa B (NF-κB) and, subsequently, its target genes, including MMP-2, MMP-9, and VEGF-A [13] (Figure 1). It has been demonstrated that aprepitant at IC_50_ concentrations of 31.55 μM and IC25 15.75 μM prevents migration of osteosarcoma cells in vitro by 80% and 42.5%, respectively. These results extrapolated to mg/kg are equivalent to approximately 20 or 10 mg/kg/day, respectively [13]. It is a well-known fact that tumor cell migration is a prerequisite for invasion and metastasis; therefore, it is logical that the use of aprepitant can prevent invasion and metastasis in osteosarcoma patients. Patients with solid tumors very often need to undergo life-saving/prolongment surgical intervention for removal of tumor mass [35,36]. However, it is known that, if a surgical insult occurs or if a biopsy collection procedure is not performed correctly, it can very often precipitate or accelerate tumor recurrence and postoperative metastases [35,37]. Furthermore, one of the mechanisms via which the SP/NK-1R system may play a role in the pathogenesis of osteosarcoma is via SP-induced migration of osteosarcoma cancer cells (which are known to overexpress NK-1R). Therefore, if SP-induced neurogenic inflammation and pain following surgical intervention are not adequately addressed, the SP/NK-1R system may possibly play an important role in osteosarcoma pathogenesis. [38]. Thus, it is crucial to find strategies aimed at reducing cancer recurrence and metastases after surgery. One of these strategies in osteosarcoma could be the use of aprepitant, a known inhibitor of invasion and metastasis of osteosarcoma cells before surgery [13]. This strategy has never ever been tested; we suggest a 10 mg/kg (IC_25_ 15.75 μM) dose of aprepitant before surgical procedures (biopsy and surgical intervention), which has demonstrated an antimetastatic effect in osteosarcoma (Figure 2). The preceding studies implicate that the use of aprepitant in osteosarcoma could possibly open the door for a new drug to be utilized in the prevention and treatment of osteosarcoma metastasis (Figure 2).

## 4. Combination of Chemotherapy and Aprepitant in Osteosarcoma Therapy

It has been reported that the combination of chemotherapy and aprepitant protects various organs and counteracts the severe side-effects of chemotherapy (cardioprotection, nephroprotection, hepatoprotection, and neuroprotection) [39,40,41,42] and cytoprotection ([4,43] (Figure 1). This combination therapy was also shown to induce tumor chemosensitization [4,5,43] (Figure 1). Additionally, it was reported in an in vitro study using MG63.2 osteosarcoma cells that the combination therapy of an NK-1R antagonist, L-733,060 compound, and different chemotherapy drugs used in the treatment of osteosarcoma (doxorubicin, cisplatin, ifosfamide, and mitomycin) increased the anticancer activity of chemotherapy (chemosensititzation) [5] (Figure 1). 

## 5. Safety and Efficacy 

The side-effects of aprepitant and fosaprepitant (a prodrug of aprepitant administered intravenously) are minimal, the most common (incidence higher than 10%) of which are constipation, fatigue, headache, anorexia, hiccups, and diarrhea [44,45,46]. The therapeutic index of aprepitant is high. By contrast, the therapeutic index of chemotherapy is extremely low (at therapeutic doses, they are cytotoxic). Chemotherapy induces severe side-effects; we and others have shown that aprepitant is safe and rather protects normal human cells, fibroblasts, nontumor HEK-293 cells, breast epithelial cells, and PC12 neurons [5,11,47]. On the other hand, adverse events associated with aprepitant have been reported in pediatric bone cancer patients. Neutropenic fever was observed at frequencies of over 44 per 100 patients, which is higher than previous estimates [48]. However, the benefits of using aprepitant far outweigh the risks, as rationalized by several studies. The damage exerted by aprepitant was tenfold higher in cancer cells than in normal leucocytes (lymphocytes) [11]. Moreover, aprepitant used at a CINV dose 125 mg/day (a concentration of approximately 4 μM) was 100 times lower than the IC50 for leukocyte toxicity. This means that aprepitant at low concentrations will presumably not produce marrow toxicity and, therefore, cannot cause neutropenic fever. 

Importantly, aprepitant is an approved drug for chemotherapy-induced nausea and vomiting (first day 125 mg; second day 80 mg; third day 80 mg). The possibility of using a 20–40 mg/kg/day dose of aprepitant in osteosarcoma therapy (as derived from extrapolating the concentration used in preclinical studies) [5,13] can be rationalized following confirmatory efficacy and safety studies for osteosarcoma. Administration of aprepitant (375 mg/day/2 weeks) to HIV patients was shown to be safe and well tolerated, leading to a decrease in the number of CD4^+^ PD-1-positive cells and SP plasma levels [49]. Moreover, since osteosarcoma cells express PD-L1, and since this expression positively correlates with tumor-infiltrating lymphocytes [50], the use of aprepitant in osteosarcoma therapy can possibly reduce the number of CD4^+^ PD-1-positive cells, leading to a decrease in the levels of osteosarcoma-infiltrating T-regulatory lymphocytes. In addition, aprepitant at 300 mg/day (for 45 days) was shown to be safe and well tolerated in patients with depression. Importantly, the side-effects of aprepitant were similar to placebo [51].

## 6. NK-1R Antagonists as Intelligent Drugs in Osteosarcoma Therapy

In the 21st century, the era of “molecularly targeted” anticancer therapy and of a “magic bullet” for cancer cells designed by Paul Erlich, NK-1R antagonists are new and promising anticancer drugs, which can be considered as a new types of “intelligent drugs” [14,47]. Conceptually, they go beyond other treatments because they display specific antitumor action. They inhibit tumor cell proliferation, induce apoptosis, and inhibit angiogenesis and migration of tumor cells for invasion and metastasis. In patients, they surprisingly provide other therapeutic benefits such as antinausea and anti-vomiting, as currently indicated and used in the clinic [52]. Furthermore, the anti-inflammatory effect of aprepitant has been demonstrated in vitro and in vivo [53], as well as in clinical trials [49,54]. Lastly, NK-1R antagonists were shown to serve as an antidepressant in clinical trials [51], in addition to leading to hepatoprotection, neuroprotection, cardioprotection, and nephroprotection in in vitro and in vivo assays [4]. Thus, aprepitant can be considered a potential therapeutic option for osteosarcoma (Figure 1).

## 7. Conclusions

Taken together, the data reported above show that NK-1R can be a potential new therapeutic target for the treatment of osteosarcoma because NK-1R antagonists (e.g., aprepitant) exert antitumor (antiproliferative, proapoptotic, antiangiogenic, antimetastatic, and tumor-shrinking) effects against osteosarcoma cells and tumors that overexpress NK-1R. In contrast, NK-1R antagonists have no detrimental effects on normal non-tumor cells because NK-1R is not essential for the viability of normal cells. The role of SP in the pathogenesis of osteosarcoma needs to be investigated in-depth using preclinical models. Furthermore, the mechanism via which SP induces pathogenesis while NK-1R antagonists protect against pathogenesis in a single setting or along with chemotherapy needs to be determined in osteosarcoma. In addition to these important studies, it must be determined whether a combination of chemotherapy and NK-1R antagonist aprepitant produces chemosensitization while decreasing the severe side-effects of chemotherapy in osteosarcoma patients. 

Thus, aprepitant, in a concentration-dependent manner, can exert a therapeutic effect as an antitumor drug against osteosarcoma due to its ability to inhibit osteosarcoma cell proliferation, induce osteosarcoma cell apoptosis, inhibit angiogenesis, and inhibit migration of osteosarcoma cells. Secondly, aprepitant can prevent and be useful in osteosarcoma metastasis. Thirdly, aprepitant must be repurposed as an antitumor drug, alone or in combination with chemotherapy in osteosarcoma therapy. Lastly, we suggest that phase I and II clinical trials are needed to assess its safety/efficacy. What are we waiting for when it could just be a question of dosage?

## Data Availability

This is a review article, where no new data were created.
